# Case Report: Three cases with allogeneic CIK therapy against solid tumors

**DOI:** 10.3389/fimmu.2025.1658864

**Published:** 2025-09-04

**Authors:** Yanfei Li, Xianwu Wang, Lili Yao, Junyan Lin, Rui Wang, Qiuhong Zheng

**Affiliations:** 1 Cell Therapy Research Center, Xiamen Humanity Hospital, Xiamen, China; 2 Department of Radiology, The Second Affiliated Hospital of Xiamen Medical College, Xiamen, China

**Keywords:** CIK, cell therapy, carcinoma, cell culture, case report

## Abstract

**Background:**

Many patients with malignant tumors fail to derive full benefits from adjuvant chemotherapy. This limitation arises from two primary factors. First, certain cancer types, such as renal cell carcinoma, hepatocellular carcinoma and cholangiocarcinoma—lack effective chemotherapy regimens. Second, factors such as advanced age, poor physical condition, and severe adverse reactions may prevent patients from completing chemotherapy. Although targeted therapies and PD-1/PD-L1 inhibitors have significantly expanded the treatment options, their efficacy depends on genetic testing results, and they remain limited by drug resistance, substantial side effects, and immune-related adverse events. In contrast, cytokine-induced killer (CIK) cell therapy involves the reinfusion of highly activated CD3+ T cells into immunocompromised patients, demonstrating a favorable safety profile without severe side effects. This approach shows significant potential for eliminating micrometastases and suppressing tumor recurrence.

**Case presentation:**

The three selected cases in this study involved malignant solid tumors, none of which had undergone standard chemotherapy. Case 1 was diagnosed with renal malignancy, for which no suitable chemotherapy regimen was available. Case 2 involved an elderly patient with advanced gastric cancer who declined chemotherapy because of concerns over its adverse effects. Case 3 was diagnosed with terminal-stage hepatocellular carcinoma, for which chemotherapy was ineffective. All of them benefited from CIK cell administration.

**Conclusion:**

We recommend the early postoperative application of CIK cell therapy for solid tumor patients, particularly in cases involving: (1) cancer types with limited chemotherapeutic options, (2) chemotherapy-intolerant patients, (3) cases of chemotherapy failure, and (4) patients who have completed standard chemoradiotherapy regimens.

## Introduction

Cytokine-induced killer (CIK) cells are derived from the *in vitro* expansion of peripheral blood mononuclear cells (PBMCs) and exhibit non-MHC-restricted cytotoxicity. The CIK cell population consists of NK-like T cells (NKT, CD3+CD56+), helper T cells (Th, CD3+CD4+CD56–), cytotoxic T lymphocytes (CTLs, CD3+CD8+CD56–), and a small proportion of natural killer cells (NK, CD3–CD56+) (1–3). Their *in vitro* cultivation requires IL-2, IFN-γ, and anti-CD3 antibody (OKT3). Since their first discovery and application in 1991 (4), CIK cells have been investigated in clinical studies for various solid tumors, demonstrating favorable efficacy and minimal side effects (5). Although CIK cell preparation has been well-established, its broader application remains limited due to inconsistent donor selection criteria, lack of standardized evaluation protocols, and the necessity for GMP-compliant production. Further clinical studies are needed to explore their combined use with chemotherapy, radiotherapy, and immune-targeted therapies. Overall, CIK cells represent a promising therapeutic approach for cancer patients (4).

## Case 1

### Case presentation

A 27-year-old male presented with a three-month history of gross hematuria prior to the initial medical evaluation in January 2019. Abdominal CT tomograph revealed a mass originating from the upper pole of the left kidney with local invasion into the renal pelvis and ureter, accompanied by pathologically enlarged retroperitoneal lymph nodes. Subsequent whole-body PET-CT not only confirmed these findings but also identified a suspicious metastatic lesion in the right clavicular region.

The patient underwent a laparoscopic radical nephrectomy on January 25, 2019. Histopathological examination confirmed a diagnosis of MIT family translocation-associated renal cell carcinoma (Xp11.2 translocation/TFE3 gene fusion) in the left kidney. The tumor, measuring 8.0 × 6.0 × 6.0 cm, demonstrated predominantly WHO/ISUP grade III morphology, with approximately 20% showing focal progression to grade IV. Pathological examination revealed areas of necrosis and local invasion of the renal pelvis and sinus fat tissue. The tumor was staged as pT3aN1Mx according to the TNM classification system. Postoperative imaging revealed an osteolytic metastatic lesion on the T12 vertebral body.

First-line therapy with sunitinib (37.5 mg qd p. o.) was initiated on February 28, 2019. On May 21, the patient underwent CT evaluation due to severe thoracic and dorsal pain (Numerical Rating Scale 6-7) that precluded supine positioning. Palliative radiotherapy (33Gy/11) was subsequently administered to the T12 vertebral lesion. Sunitinib was discontinued on June 11, following the development of grade 2 cutaneous toxicity, manifesting as an erythematous rash. Restaging PET-CT revealed disease progression characterized by (1) new retroperitoneal lymph node metastases (2) additional osteolytic lesions in the thoracic vertebrae and (3) progression of a previously identified clavicular lesion. PFS1: 4 months.

Between June 18, 2019, and August 7, 2020, the patient received combination therapy consisting of axitinib (5 mg bid p. o.), pembrolizumab (200 mg d1/q3w ivgtt.) and zoledronic acid (4 mg q4w ivgtt). From May 11 to 22, 2020, palliative radiotherapy (40Gy/10) was administered to the metastatic lesions in the right iliac bone and T7-T8 vertebrae. By September 2020, the patient had developed progressive bilateral lower extremity weakness (right more affected than left) accompanied by neurogenic bladder dysfunction. Subsequent PET-CT imaging confirmed the disease progression. PFS2: 16 months.

The patient received a combination therapy with tislelizumab (200 mg ivgtt d1) and axitinib (5 mg bid p. o.) in 3-week cycles from December 9, 2020, to July 13, 2021. In mid-July 2021, the patient developed new-onset neurological symptoms including dizziness, nausea, and vomiting. Subsequent PET-CT imaging revealed disease progression with new metastatic lesions in the right cerebellar hemisphere and newly enlarged lymph nodes along the para-aortic region. PFS3: 7 months.

From July 2021 to June 2022, the patient underwent two courses of carbon ion radiotherapy (40Gy/5) targeting progressive brain metastases and enlarged para-aortic lymph nodes. Concurrently, the patient was administered oral cabozantinib (40 mg qd p. o.) as systemic therapy. PFS4: 7 months.

The patient initiated fifth-line therapy on June 20, 2022, with pembrolizumab (200mg d1/q3w ivgtt). and lenvatinib (8mg qd po.). By November 2022, disease progression was evident on imaging and brain MRI demonstrated enlargement of the right cerebellar and temporal lobe lesions, while PET-CT revealed thickening and hypermetabolism of the right obturator internus muscle alongside a new T4 vertebral metastasis. Over the subsequent months, progression was observed in the abdominal/iliac lymph nodes, T4 lesion, and obturator internus muscle, with a new metastatic nodule in the lingular segment of the left upper lung. These received carbon ion radiotherapy (abdominal/iliac nodes 52.8 Gy/12; T4 26.4 Gy/6; lung 72 Gy/9). Another two cycles of adjunctive bevacizumab (15 mg/kg q3w ivgtt) were administered in May and October 2023, with the treatment concluded in November 2023. PFS5: 16 months.

The patient had been receiving combination therapy with cadonilimab (initially 625 mg d1/q3w ivgtt), which was subsequently reduced to 500 mg d1/q4w for improved tolerability) and lenvatinib (8 mg qd p. o.) from November 23, 202, at Xiamen Hong’ai Hospital. In addition to this ongoing systemic treatment, four cycles of bevacizumab (300 mg d1/q2w ivgtt.) was administered as adjunctive therapy in April and May 2024. Since March 2024, the treatment plan has been supplemented with comprehensive supportive care including traditional acupuncture, infrared therapy, joint mobilization exercises, low-frequency pulsed functional electrical stimulation, and occupational therapy to optimize clinical outcomes.

### The start of CIK therapy

**Figure 1:**
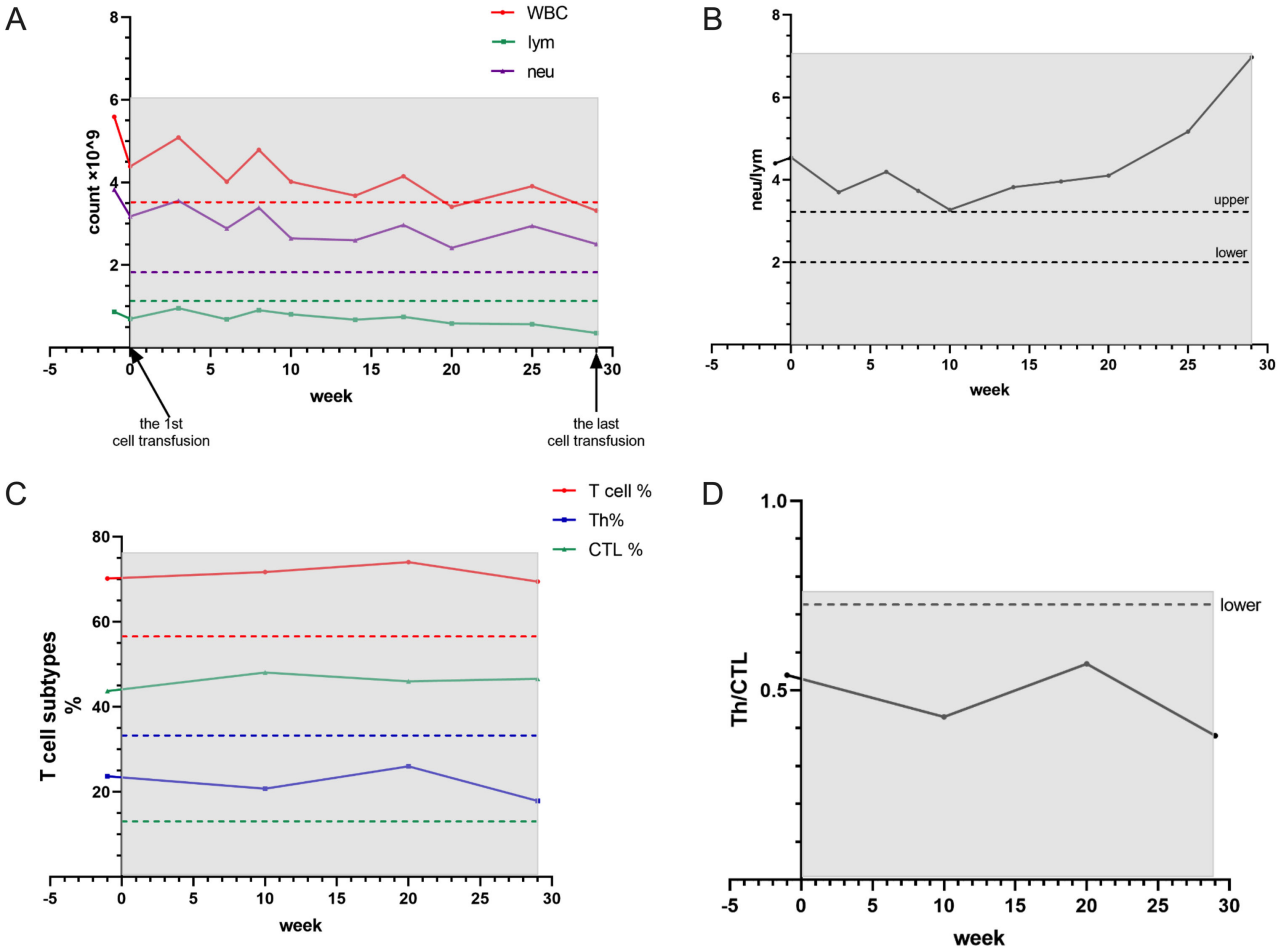
The shaded region spans the entire duration of cell transfusion therapy. Reference ranges are indicated by dashed lines, representing the lower limit of normal values. Lymphocyte counts remained consistently below the reference range despite intermittent elevations **(A)**. The neutrophil-to-lymphocyte ratio (NLR) exhibited an accelerated increase beginning at week 20, corresponding to the 8th cycle of cell therapy **(B)**. CD4+ T helper (Th) cell percentages persisted below the normal reference range, while cytotoxic T lymphocyte (CTL) levels maintained elevated values throughout the observation period **(C)**. This inverse relationship resulted in a sustained reduction in the Th/CTL ratio **(D)**.

**Figure 2:**
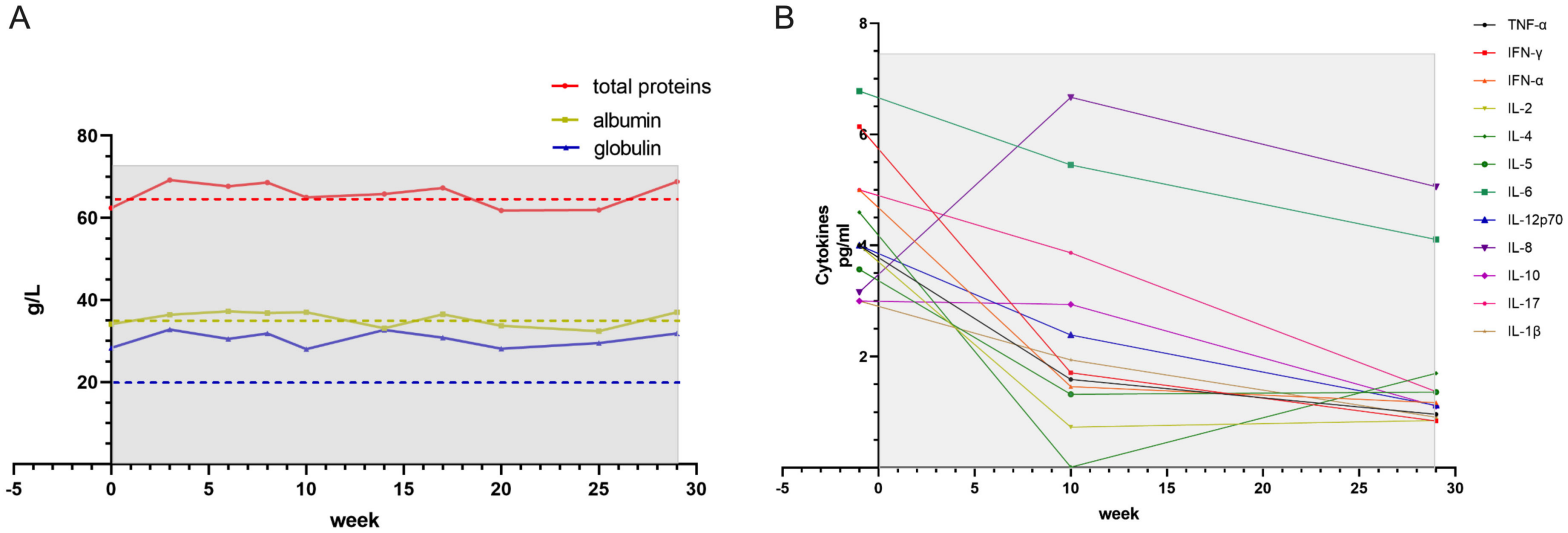
Serum protein evaluation demonstrated maintenance of total protein and albumin levels near the lower normal limit while globulin concentrations remained at the upper normal range **(A)**. Cytokine profile following CIK therapy revealed significant reductions in most measured cytokines, with the notable exceptions of persistently elevated IL-2, IL-4 and IL-5 levels **(B)**.

The patient underwent CIK cell therapy at the Xiamen Hong’ai Hospital beginning in November 2024, with the final cell transfusion completed on June 5, 2025. The therapy utilized allogeneic peripheral blood mononuclear cells obtained from the patient’s mother. Ten fresh cell transfusions were successfully performed without complications. Representative laboratory findings throughout the treatment course are shown in **Figures 1A–D** and **Figures 2A, B**. In **Figure 1A**, the leukocyte count fluctuated in response to cell transfusion, and a similar trend was observed for neutrophils. Given that lymphocytes constitute a smaller proportion of peripheral blood compared to neutrophils, their numerical variations were less pronounced. Overall, lymphocyte levels exhibited a gradual decline. **Figure 1B** demonstrates that the neutrophil-to-lymphocyte ratio (NLR) began to increase at 10 weeks post-infusion, indicating a more rapid decline in lymphocytes relative to neutrophils. **Figures 1C, D** reveal that lymphocyte counts remained above the lower limit of normal, with CTLs predominating, while Th persisted at consistently low levels. **Figure 2A** shows stable levels of total protein, albumin, and globulin, suggesting adequate nutritional status and normal B-cell function. **Figure 2B** depicts the peripheral blood cytokine profile: IL-6, IL-17, IL-12p70, IL-1β, TNF-α, IFN-γ, and IFN-α exhibited a sustained decline, and of them, IL-6 and IL-17 followed nearly identical trends.IL-12p70 and IL-1β shared a similar pattern. TNF-α, IFN-γ, and IFN-α displayed closely aligned dynamics.IL-10 began to decrease at 10 weeks after initial cell therapy.IL-2, IL-4, and IL-5 initially declined before gradually rebounding. Only IL-8 showed an initial increase followed by a subsequent decrease. Although surveillance imaging demonstrated gradual enlargement of the abdominal and iliac lymph nodes, the patient maintained stable disease status without evidence of systemic progression. Notably, the patient achieved sustained clinical stability with preserved quality of life throughout the treatment period.

### Patient perspective

Prior to initiating CIK cell infusion therapy, the patient reported he had experienced multiple debilitating symptoms in daily life, including insomnia, loss of appetite, irritability, and increased susceptibility to colds and fever. Remarkably, significant clinical improvement was observed following the first CIK infusion, including marked enhancement in sleep quality, gradual improvement in appetite with subsequent weight gain, increased physical strength enabling more intensive rehabilitation training, complete absence of influenza or common cold infections during the 2025 winter and spring seasons following treatment. All these responses indicated substantial improvement in the patient’s quality of life following CIK cell therapy.

## Case 2

### Case presentation

**Figure 3:**
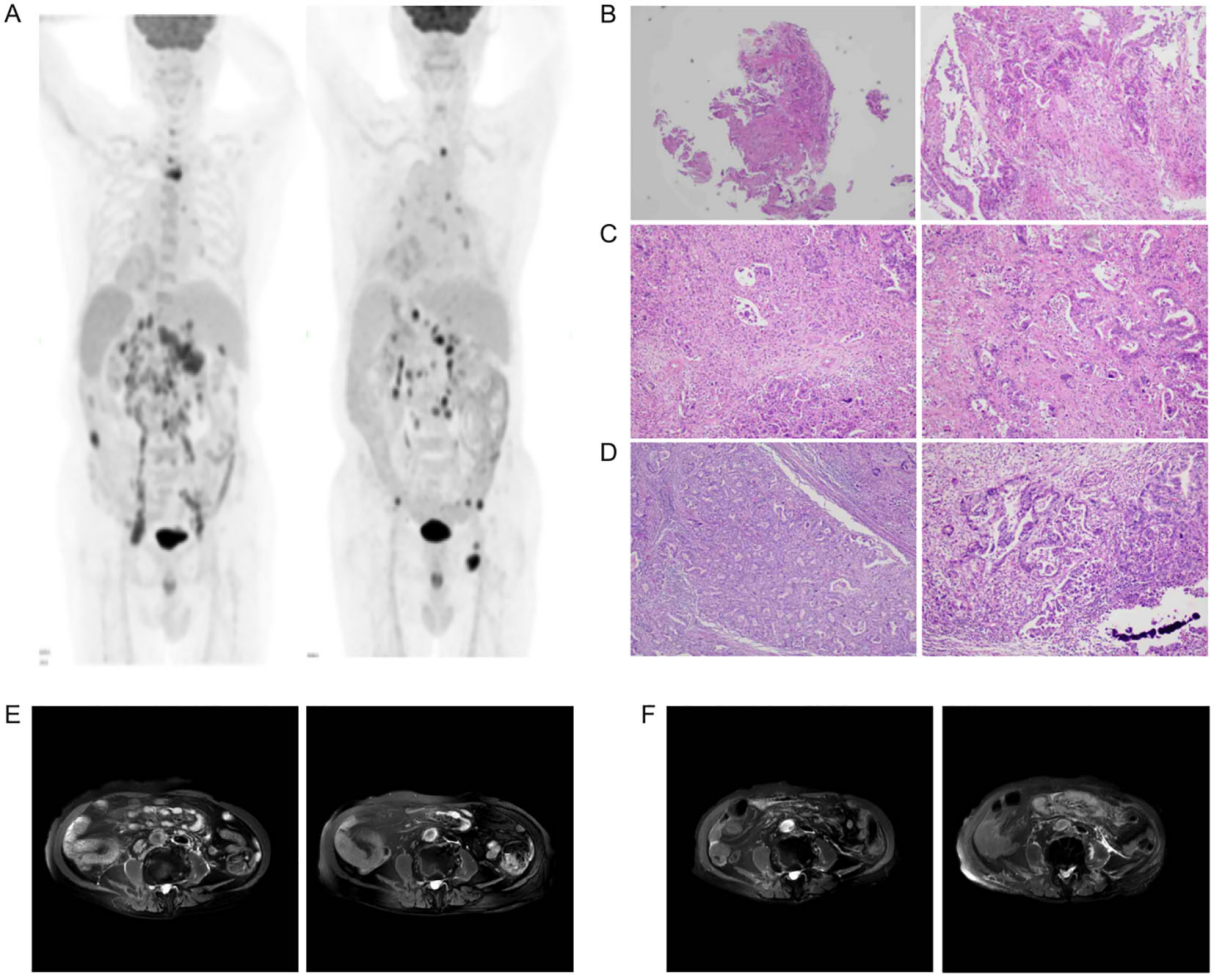
PET-CT [*2023-11 left vs 2024-04 right*, **(A)**]. Endoscopic biopsy from gastric antrum and duodenal bulb under endoscope [2023-11, **(B)**, *left×100, right×400 right*]. Hepatic lesion resection [2023-11, **(C)**, *left×100, right×400 right*]. Left inguinal lymph node [2024-04, **(D)**, *left×100, right×400 right*]. Abdominal MRI T2 [*2023-10 left vs 2024-01 right*, **(E)**]. Abdominal MRI T2 [*2024-06 left vs 2024-12 right*, **(F)**].

An 85-year-old male with a history of chronic kidney disease (8-year duration), normocytic anemia (3-year duration), well-controlled hypertension, and asymptomatic hyperuricemia (4-year duration) was admitted on October 30, 2023, for the evaluation of persistent abdominal distension lasting one month. The patient, a former smoker with a 50 pack-year history (cessation 5 years prior), reported no additional symptoms. Diagnostic imaging revealed an abdominal CT demonstrating a space-occupying lesion in the gastric antrum with suspected metastatic involvement of the porta hepatis, abdominal cavity, retroperitoneum, and bilateral iliac fossa lymph nodes; PET-CT (November 3, 2023; **Figure 3A**) confirmed situs inversus totalis, gastric antral carcinoma with extensive lymph node metastases (porta hepatis, retroperitoneal, and iliac), vertebral metastases (C7 and T3), and multifocal hepatic metastases. Endoscopic biopsy obtained six approximately 1.0 mm specimens from the gastric antrum and duodenal bulb. Histopathological examination (**Figure 3B**) identified moderately differentiated adenocarcinoma with an immunohistochemical profile consistent with a gastrointestinal origin, showing features suggestive of either gastric-type or pancreatobiliary-type differentiation.

The patient declined to receive systemic chemotherapy. Palliative radiotherapy was administered to C7 and T3 vertebral metastases (20Gy/5) from November 13 to 17. On November 19, the patient developed postprandial vomiting of undigested food, which was clinically consistent with a gastric outlet obstruction secondary to advanced carcinoma. Emergency laparoscopic procedures were performed on November 20, including gastrojejunostomy for palliation of the obstruction and resection of the dominant hepatic lesion. Histopathological examination of the hepatic lesion (**Figure 3C**) confirmed moderately differentiated adenocarcinoma with lymphovascular invasion. The immunohistochemical profile was most consistent with a primary duodenal or pyloric canal origin. Final staging was classified as TxN+M1 (Stage IV) according to the AJCC 8th edition criteria.

### The start of CIK therapy

Combination therapy with pembrolizumab (200 mg d1/q3w ivgtt) was initiated and CIK cell therapy on November 10, 2023, using allogeneic peripheral blood mononuclear cells from the patient’s son. Serial PET-CT imaging in April 2024 (**Figure 3A**), compared with baseline (November 3, 2023), demonstrated a significant reduction in size and metabolic activity of the primary gastric antral lesion regression or complete resolution of most metastatic lymph nodes, although with progression in the mesenteric root and new involvement in the left inguinal region and bilateral iliac vasculature absence of detectable hepatic metastases and development of osteoblastic changes in the previously irradiated C7 and T3 vertebral metastases. Pathological confirmation of the left inguinal lymph node metastasis (**Figure 3D**) revealed adenocarcinoma consistent with the primary duodenal-pyloric canal tumor. The treatment course was complicated by pembrolizumab-induced immune-related pneumonitis after seven cycles, necessitating discontinuation on May 9, 2024. CIK therapy was continued until August 9, 2024, with 10 uneventful cell transfusions completed. All antineoplastic therapies were subsequently discontinued with a transition to purely supportive care. Serial imaging revealed marked regression of the abdominal and retroperitoneal lymph nodes in January 2024 after the third transfusion performed (**Figure 3E**, *2023-10 left vs 2024-01 right*). However, when he stopped receiving CIK transfusion disease for about 4 months, when found disease progression in December 2024 compared with MRI scan around the ninth transfusion (**Figure 3F**, *2024-06 left vs 2024-12 right*).

**Figure 4:**
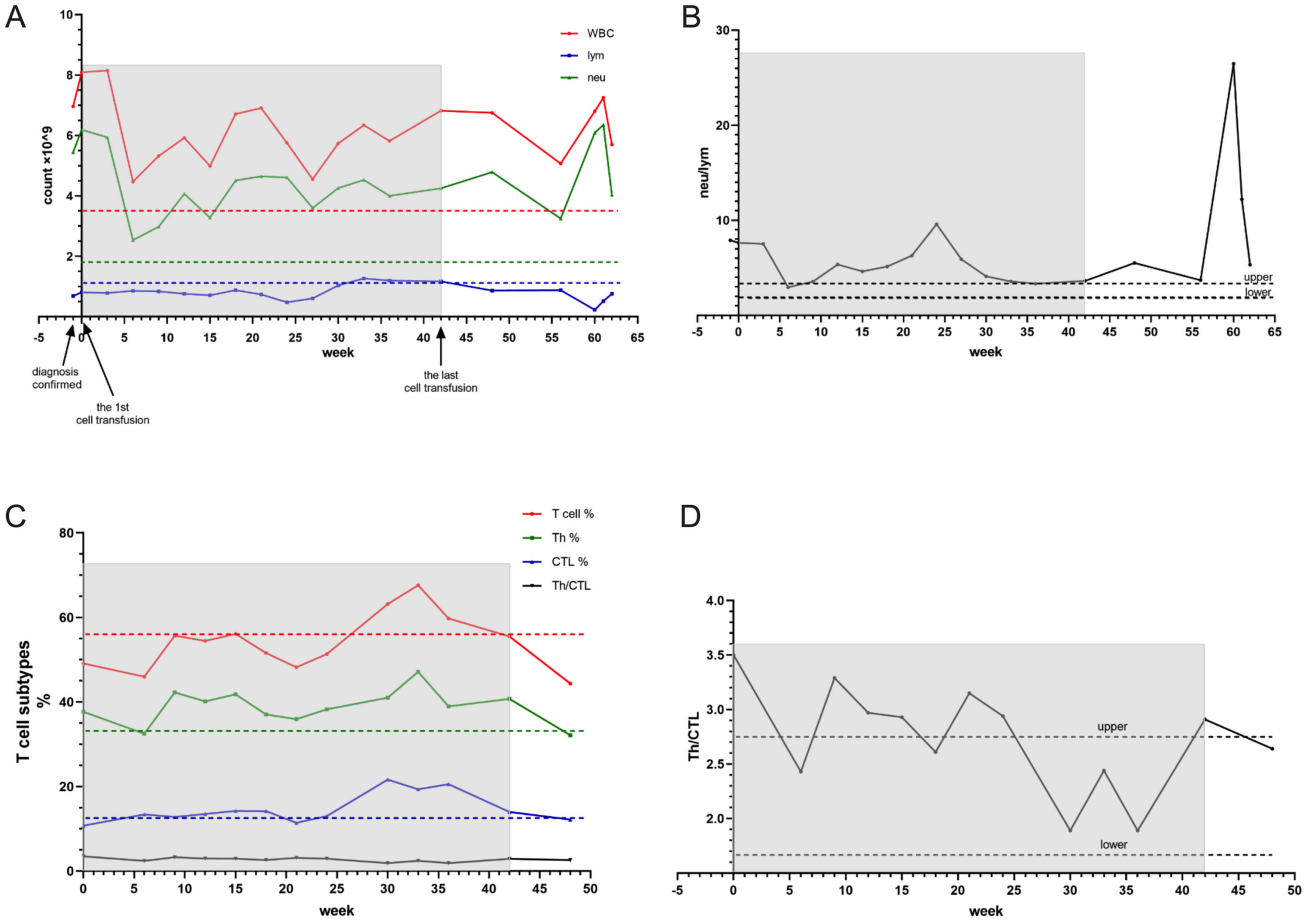
The light shadow covers the beginning till the ending of cell transfusion. The dash line represents the lower value of the test reference. At the beginning of CIK therapy the lymphocytes maintained at lower value and it rise after the 30th week i.e. the 8th transfusion **(A)**. The NLR also illustrated the similar result **(B)**. T-helper subgroup dominates in the total T cells and Th/CTL never comes down below the lower reference value **(C, D)**.

**Figure 5:**
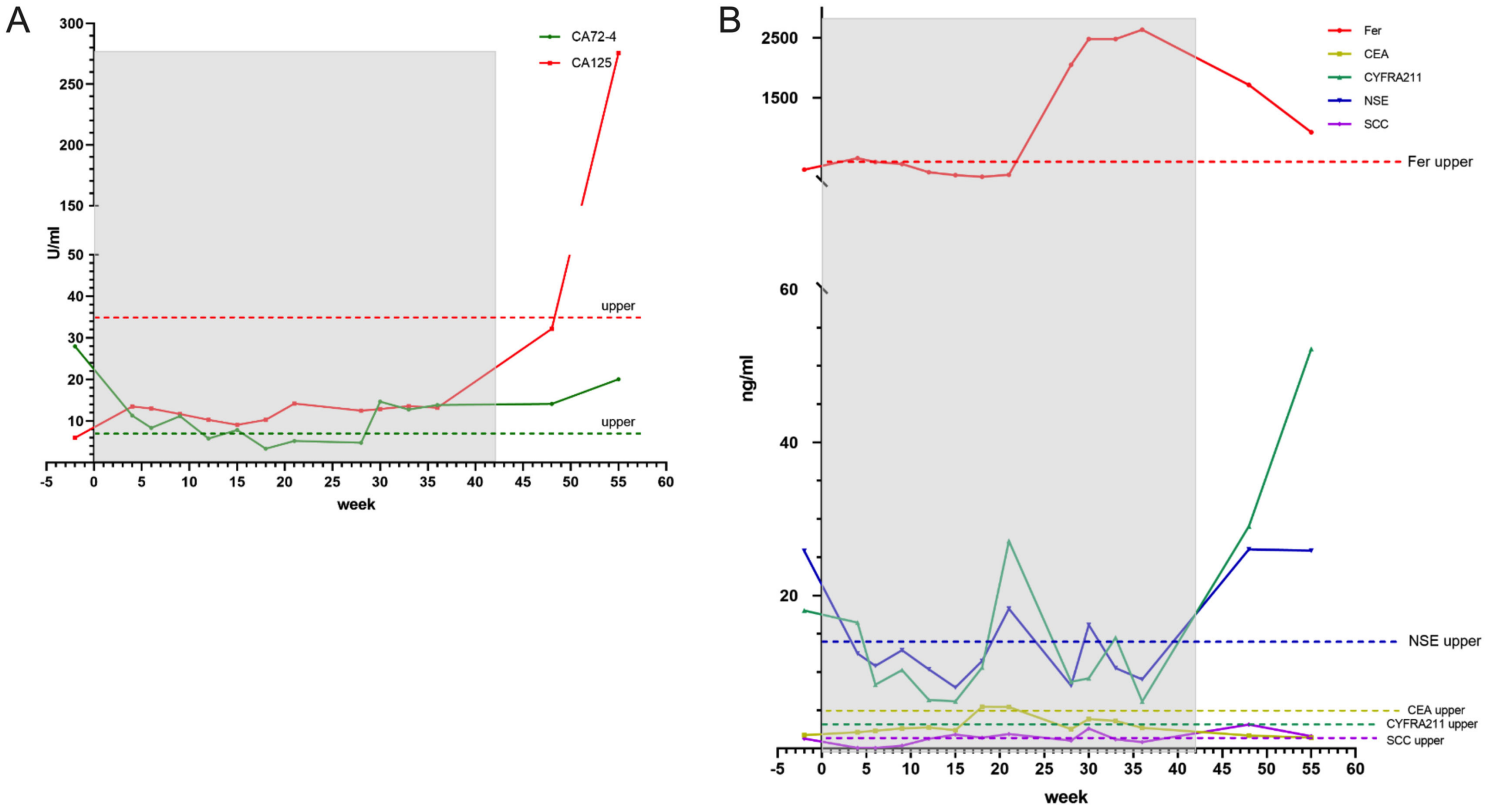
Among the various tumor markers, CA72-4, CA125, NSE and CYFRA211 fluctuate more obviously than others. When CIK therapy come into end, they rise swiftly **(A, B)**.

Serial imaging surveillance revealed disease progression in December 2024, with abdominal CT demonstrating significant advancement of the primary gastric lesion. Subsequent thoracic CT in February 2025 revealed multiple metastatic pulmonary lesions. The patient ultimately succumbed to complications such as pulmonary infection and sepsis. Representative laboratory parameters monitored throughout the course of CIK cell therapy are presented in **Figures 4A–D** and **Figures 5A, B**, documenting key hematologic and immunologic trends during treatment. Following two CIK cell infusions, peripheral leukocyte and neutrophil counts demonstrated a significant decline (**Figure 4A**), while lymphocyte levels remained stable. After seven infusion cycles, lymphocytes increased to within normal range and maintained this level for approximately 10 weeks. Cessation of therapy was followed by a subsequent lymphocyte decrease. Notably, lymphocyte counts reached their nadir coinciding with pulmonary infection (approximately 13 weeks post-treatment discontinuation), while neutrophils and the NLR peaked simultaneously (**Figure 4B**). T-cell subtypes revealed consistently higher Th than CTL proportions (**Figures 4C, D**), a pattern distinct from Case 1. The absolute T-cell count peaked at the seventh CIK infusion. CA 72-4 showed rapid initial decline post-infusion, followed by fluctuation near upper reference limits. CA 125 remained below cutoff values throughout treatment, with both markers exhibiting gradual elevation after therapy cessation (**Figure 5A**). Ferritin levels increased during active treatment phases, temporally correlating with T cell increase (**Figure 5B**). CYFRA 21-1 and NSE fluctuated near upper normal limits during therapy but rose following treatment discontinuation. In contrast, SCC and CEA remained stable throughout the observation period (**Figure 5B**). In December 2024, abdominal CT revealed the progression of the stomach. In February, 2025, thoracic CT revealed multiple metastatic lesions in the lungs. The patient died of a pulmonary infection and sepsis one month later.

### Patient perspective

The patient was an elderly male who explicitly declined conventional chemotherapy due to concerns about treatment-related adverse effects. With a documented smoking history, he developed immune-related pneumonitis following immunotherapy. Consequently, while maintaining carefully titrated doses of immune-targeted agents, the patient was initiated on cellular therapy. Notably, the cell therapy regimen was well-tolerated throughout the treatment course, with no treatment-emergent adverse events appeared, no disruption to sleep patterns or dietary habits and an improvement in daily activity levels.

## Case 3

### Case presentation

**Figure 6:**
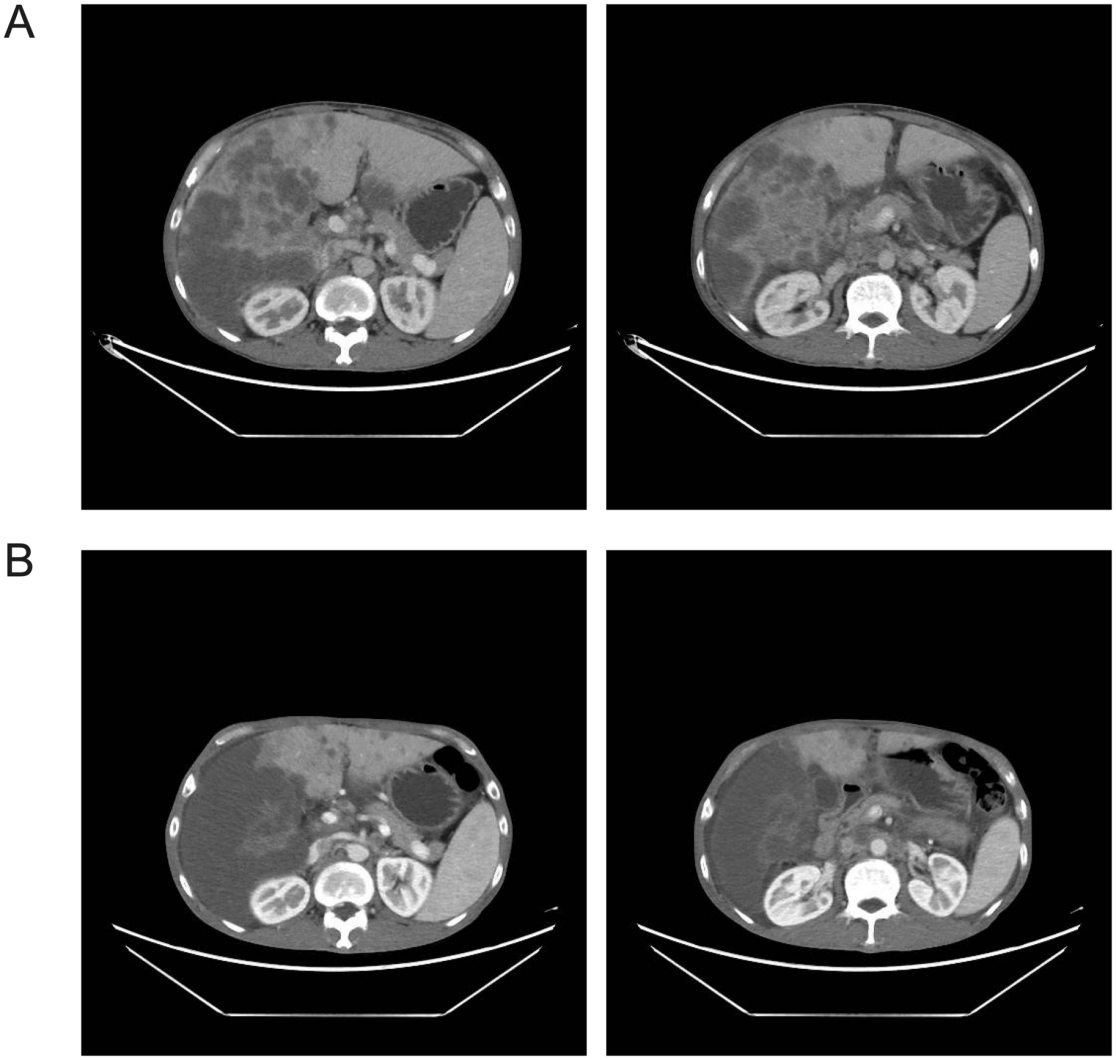
Abdominal CT (A 2024-2-20 vs B 2024-04-22). The liver demonstrates diffuse nodular and mass-like hypodense lesions, with the largest cross-sectional dimension measuring approximately 4.34 cm × 3.49 cm. Some lesions show coalescence with ill-defined margins, predominantly in the right hepatic lobe. Multiple small retroperitoneal lymph nodes are noted **(A)**. The hepatic parenchymal masses and nodules have further coalesced into conglomerates with decreased density compared to prior imaging. New hypodense nodules are noted in the left hepatic lobe with increased number relative to previous examination. No significant change is observed in the ascites within the peritoneal and pelvic cavities **(B)**.

A 48-year-old male presented to our institution on February 20, 2024, with a three-month history of progressive weight loss and fatigue. Abdominal CT imaging (**Figure 6A**) revealed the following significant findings: multiple hepatic masses with radiographic features suggestive of malignant lesions, most consistent with metastatic disease, numerous small retroperitoneal lymph nodes, moderate ascites with concomitant pelvic fluid accumulation, multiple pulmonary nodules suggestive of metastatic spread, and minimal bilateral pleural effusions. On February 22, the patient underwent ultrasound-guided percutaneous liver biopsy and therapeutic paracentesis. Histopathological examination confirmed the diagnosis of intrahepatic cholangiocarcinoma. A multidisciplinary tumor board review determined that lesions were unresectable owing to the extent of metastatic involvement. The patient initiated first-line hepatic arterial infusion chemotherapy of oxaliplatin (85 mg/m², day 1) and gemcitabine (1000 mg/m², day 8) on February 28, 2024. Cytological examination of the ascitic fluid on March 8 showed no evidence of malignant cells. However, a follow-up CT on March 18 demonstrated disease progression, characterized by an increased number and size of hepatic metastatic lesions. Second-line therapy with toripalimab (240 mg d1/q3w ivgtt), and lenvatinib (8 mg qd p. o.) were initiated.

### The start of CIK therapy

**Figure 7:**
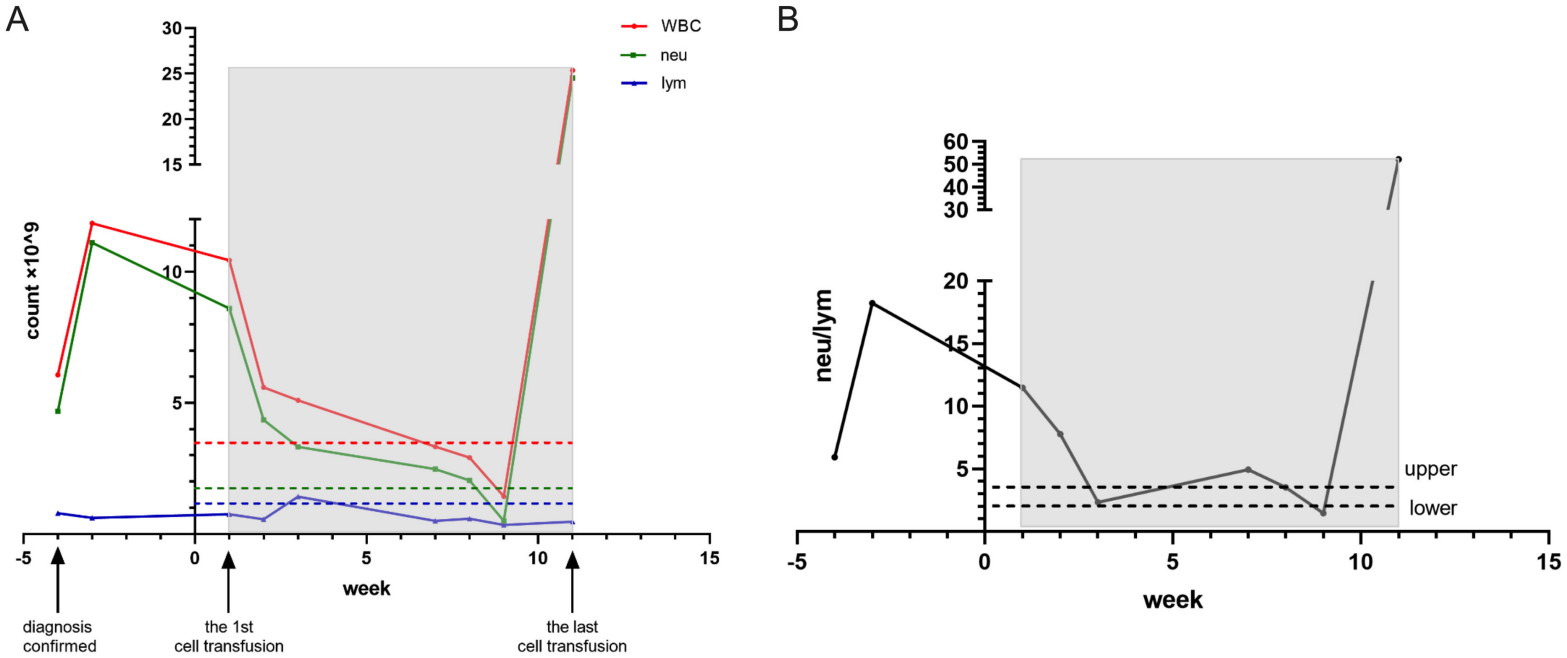
The light shadow covers the beginning till the ending of cell transfusion. The dash line represents the lower value of the reference. The neutrophils come down rapidly after first CIK transfusion and lymphocytes rise slowly. After the 6th cell therapy the neutrophils rise sharply and lymphocytes keep at a very low level **(A)**. The same result represents in NLR **(B)**.

**Figure 8:**
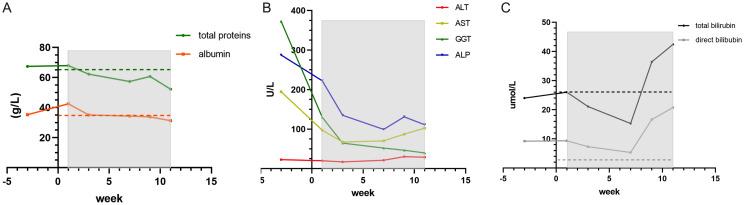
The total proteins and albumin maintain at a nearly normal range **(A)**. ALT, AST, GGT and ALP all come down after first CIK therapy **(B)** The total and direct bilirubin drop at first place and rise again at 5th cell therapy **(C)**.

**Figure 9:**
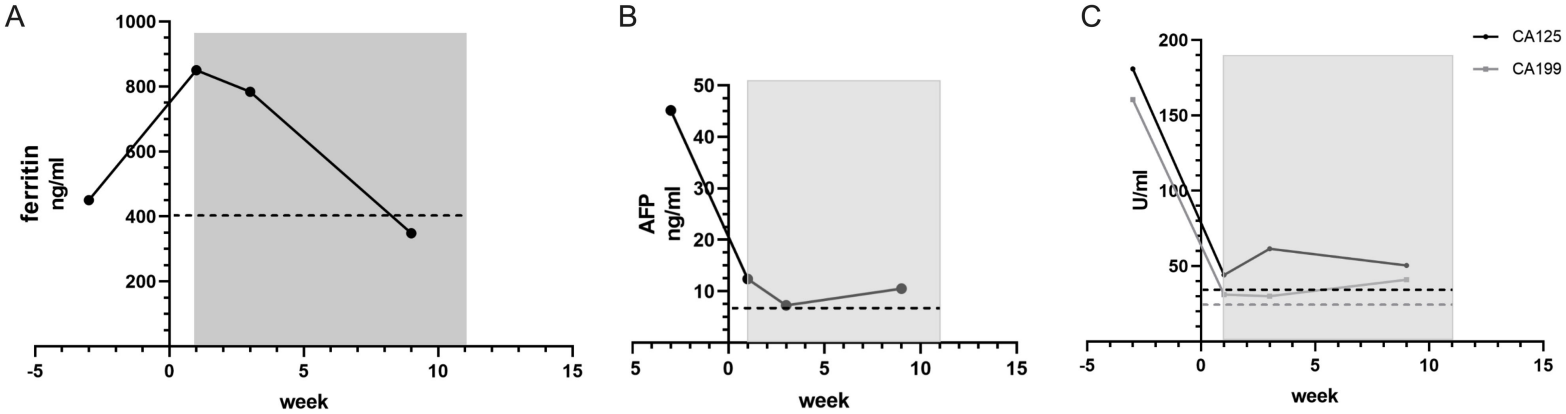
Ferritin descends when CIK therapy begins **(A)**. AFP, CA125 and CA199 are all drop before CIK intervention and are kept on a steady low state **(B, C)**.

Concurrently, CIK cell therapy was administered the following day, using allogeneic peripheral blood mononuclear cells from the patient’s son. Repeat CT evaluation on April 22 revealed decreased density of multiple intrahepatic metastases, reduced ascites and pleural effusions, and mild progression of retroperitoneal lymphadenopathy (**Figure 6B**). The patient continued triprolizumab and lenvatinib until May 22, and CIK therapy was completed on June 3, 2024 (7 transfusions without complications). Representative laboratory parameters during CIK therapy are presented in **Figures 7A B**, **Figures 8A–C** and **Figures 9A–C**. At initial diagnosis, the patient presented with ascites, pelvic effusion, and concurrent infection, accompanied by elevated leukocyte and neutrophil counts that showed modest reduction following anti-infective therapy. Subsequent CIK cell infusion elicited marked hematological changes, with leukocytes and neutrophils decreasing to normal ranges within 2 weeks post-infusion, though these parameters rebounded sharply when abdominal infection became uncontrolled at week 9. Lymphocyte counts demonstrated gradual normalization followed by subsequent decline (**Figure 7A**), a pattern mirrored by the NLR (**Figure 7B**). Concurrently, progressive declines in total protein and albumin reflected worsening nutritional status (**Figure 8A**), while hepatic function tests revealed sustained decreases in GGT and ALP alongside initially significant reductions in AST, ALT, total and direct bilirubin that subsequently began gradual increases from week 7 (**Figures 8B, C**). Tumor marker surveillance showed consistent downward trends in ferritin, AFP, CA125 and CA19-9 throughout the treatment period (**Figures 9A–C**). The treatment was subsequently transitioned to best supportive care and the patient died of peritonitis and sepsis ultimately one month later.

### Patient perspective

The patient presented at diagnosis with significant cachexia, fatigue, abdominal distension, anorexia, and sleep disturbances. Upon learning that curative surgery was not feasible, he became profoundly depressed and expressed a strong preference to avoid additional treatment-related adverse effects, hoping instead for a peaceful end-of-life experience. Remarkably, clinical improvements were observed following CIK cell infusion, with sleep quality enhancing within 24 hours post-infusion. By one week after treatment, the patient exhibited reduced abdominal distension, improved appetite, and regained the ability to ambulate independently for one hour daily.

## A brief summary

**Figure 10:**
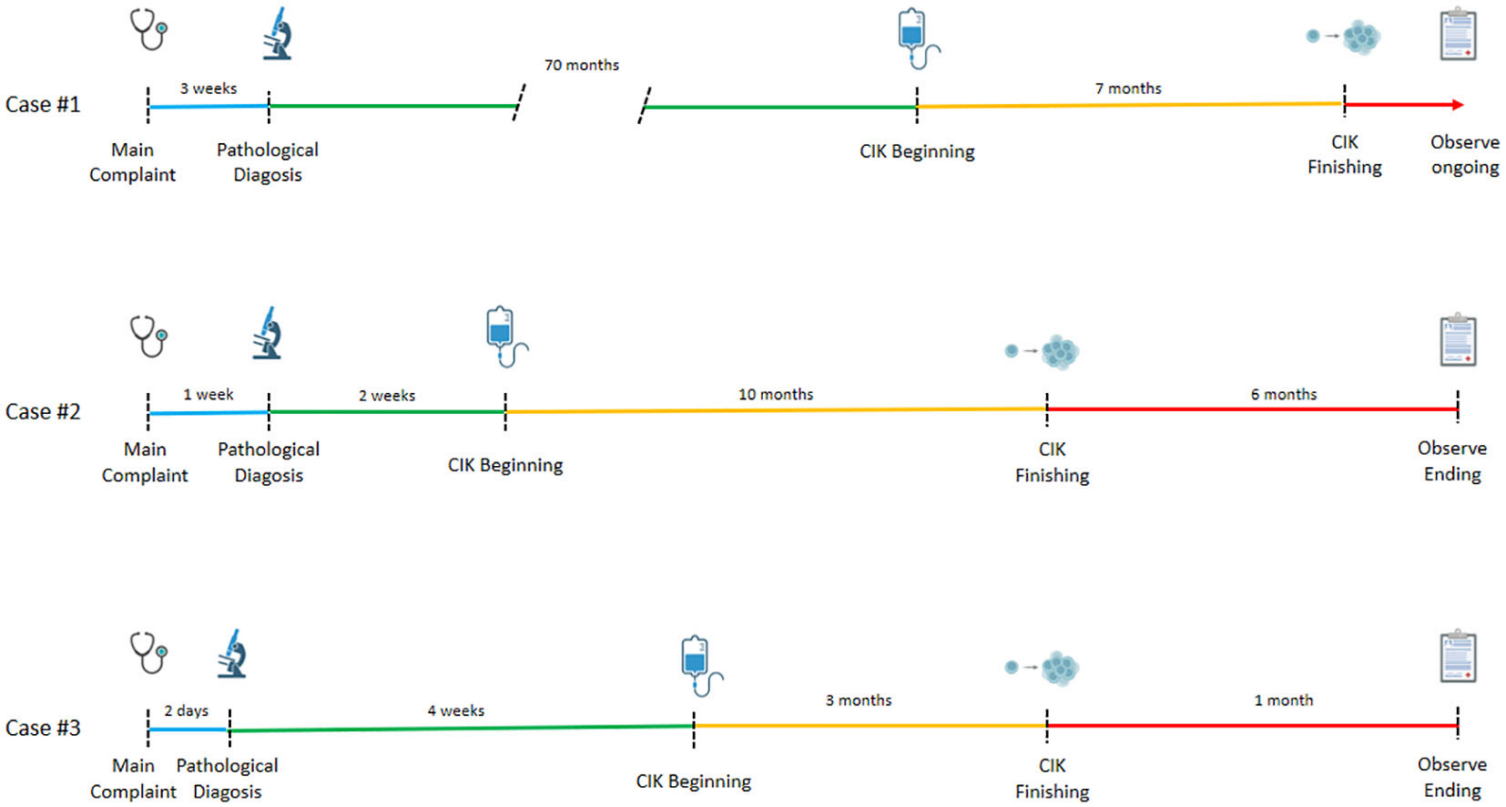
A retrospective timeline of the three cases.

The three cases are summarized in briefly in **Figure 10** and **Table 1**.

**Table 1:** A brief summary of the three cases.

Case Number	Age/sex	Main complaint	Pathological diagnosis	Chemotherapy via vein	Targeted medicine	PD-1/PD-L1/CTLA-4 inhibitor	CIK duration/ times	Observe ending
Case 1	27/male	Hematuria	Renal cell carcinoma	N/A	Sunitinib Axitinib Cabozantinib Lenvatinib Bevacizumab	Pembrolizumab Tislelizumab Cadonilimab	29 weeks/10	N/A, another course of CIK is going on
Case 2	85/male	Abdominal distension	Gastric adenocarcinoma	N/A	None	Pembrolizumab	42 weeks/10	Pulmonary infection and sepsis
Case 3	48/male	Weigh loss/fatigue	Intrahepatic cholangiocarcinoma	N/A	Lenvatinib	Toripalimab	11weeks/7	Peritonitis and sepsis

## Discussion

The three selected cases in this study involved malignant solid tumors, none of which had undergone standard chemotherapy. Chemotherapy refusal or ineligibility represents a frequently encountered clinical scenario. Case 1 was diagnosed with a renal malignancy, for which no suitable chemotherapy regimen was available. Case 2 involved an elderly patient with advanced gastric cancer who declined chemotherapy because of concerns over its adverse effects. Case 3 was diagnosed with terminal-stage hepatocellular carcinoma, for which chemotherapy was ineffective; only one cycle of hepatic arterial infusion chemotherapy was administered for palliative symptom relief.

Case 1 involved a young male patient with prolonged progression of renal carcinoma progression. Despite undergoing multiple lines of postoperative targeted immunotherapy and radiotherapy, the tumor exhibited slow progression. Upon initial admission, the patient presented with fatigue, insomnia, and progressive weight loss accompanied by diminished confidence in pharmacotherapy. Following four cycles of cytokine-induced killer (CIK) cell therapy, the patient demonstrated symptomatic improvement in fatigue and insomnia, increased appetite, and stabilization of laboratory parameters, including leukocyte count, lymphocyte count, and T-cell subset proportions (**Figures 1A, C**).

The overall trend of cytokine profile revealed elevated levels of IL-2, IL-4, IL-5 (which stimulate B- and T-cell proliferation, enhance macrophage activity, and promote immunoglobulin maturation) and IL-8, (mediates neutrophil chemotaxis and activation). In contrast, pro-inflammatory cytokines (TNF-α, IFN-γ, IFN-α, IL-1β, IL-6, IL-12 and IL-17) and immunomodulatory cytokines (IL-10) were significantly reduced (**Figure 2B**), suggesting enhanced immune function with subsequent stabilization (6, 7). More specifically, both IL-6 and IL-17 serve as pivotal proinflammatory factors (8) that are markedly upregulated during early inflammation. Notably, IL-6 potentiates Th17 differentiation and subsequent IL-17 secretion (9), establishing a mechanistic link that explains their parallel decline. The observed reduction in IL-1β similarly reflects attenuated inflammatory responses. While IL-12 drives Th1 differentiation and Th1 secretes IFN-γ production, IFN-γ exhibiting dual pro-inflammatory and antitumor effects (10). The coordinated decrease in IFN-α, IL-12, and IFN-γ may indicate either T-cell exhaustion (11) or tumor immune evasion (12). The decline in TNF-α likely results from diminished tumor burden and consequent reduction in chronic inflammation (13). IL-10, possessing immunomodulatory properties, requires comprehensive evaluation of both malignant and immune cell activities for proper interpretation; its reduction suggests waning immunosuppression (potentially indicating impaired host immunity) while paradoxically possibly reflecting amelioration of tumor-associated chronic inflammation. IL-8 demonstrated unique biphasic kinetics (initial elevation followed by decline), likely attributable to decreased chemotactic stimulation of neutrophils as tumor microenvironment activity subsided (14). The cytokines IL-2, IL-4, and IL-5 shared characteristic kinetic profiles (initial decrease followed by gradual increase), with IL-4 showing most pronounced recovery. Functionally, IL-4 promotes Th2 differentiation and IgG production (in concert with IL-5) while suppressing Th1 responses via IL-12 downregulation (15). IL-2 enhances lymphocyte (T/B/NK cell) activity (16), whereas IL-5 stimulates B-cell differentiation (17). The initial decline may reflect systemic immune exhaustion, while subsequent recovery potentially represents therapeutic effects of CIK cell infusion. Although recent imaging detected persistent enlargement of the abdominal and retroperitoneal lymph nodes, these findings did not compromise the patient’s activities of daily living (18).

Case 2 involved an elderly male patient who presented with widespread metastases at initial diagnosis. Given his ineligibility for conventional chemotherapy, targeted immunotherapy was promptly initiated after diagnosis. Considering his advanced age and frailty, cytokine-induced killer (CIK) cell therapy was incorporated into the treatment regimen. Remarkably, after just one cycle of CIK cell infusion, the patient achieved sufficient clinical improvement to undergo laparoscopic surgery and hepatic lesion resection and significant regression of the systemic lesions was observed (**Figures 3A, E**). Although formal stable disease (SD) status was not attained, the patient maintained normal appetite, sleep patterns, and bowel/bladder function without experiencing chemotherapy-associated toxicities. During the infusion period, lymphocyte counts and T-cell subsets remained within physiological ranges (**Figures 4A, C**). Following CIK cell infusion, a significant decline in both leukocyte and neutrophil counts was observed, indicative of restored immune function and reduced acute inflammation. Repeated administrations led to lymphocyte levels increasing to within normal ranges, where they remained stable for approximately 10 weeks before gradually declining after treatment cessation. Notably, comparative analysis revealed distinct T-cell subset distributions between cases-while case 2 maintained a consistently higher Th to CTL ratio (**Figure 4C**), Case 1 demonstrated the opposite pattern with CTL predominance (**Figure 1C**). This immunological divergence may stem from either: (1) inherent variations in the CTL/Th composition of donor-derived CIK cell products (**Supplementary Figure 2**), or (2) patient-specific factors including age, baseline immune status, and tumor type characteristics.

Notably, two lung cancer-specific biomarkers, neuron-specific enolase (NSE) and cytokeratin 19 fragment (CYFRA21-1), persisted at abnormally elevated levels (**Figure 5B**), potentially reflecting the gastric tumor’s dual epithelial origin and neuroendocrine differentiation features of the gastric tumor. Emerging evidence indicates that CYFRA21-1 demonstrates the highest sensitivity for gastric cancer detection, while NSE and CA72-4 serve as reliable markers for predicting lymph node involvement (19, 20). In this context, the gradual elevation of CA72-4, NSE, and CYFRA21-1 levels observed around week 30 of CIK treatment (**Figures 5A, B**) may suggest early disease progression. Although ferritin exhibits limited specificity as a tumor marker (21), its transient elevation at week 20 post-CIK infusion-temporally coinciding with peak T-cell expansion, potentially reflects extensive tumor cell apoptosis/necrosis with subsequent release of cellular contents into circulation, followed by gradual normalization. Following CIK therapy discontinuation, progressive immunological decline was observed, characterized by decreased lymphocyte and T cell counts. Concurrently, tumor markers began to increase progressively until disease progression culminated in multiorgan failure and systemic infection. Emerging therapeutic strategies combining chemotherapy demonstrate enhanced antitumor efficacy in advanced gastrointestinal cancer (22, 23).

Case 3 involved a middle-aged male patient diagnosed with terminal-stage hepatocellular carcinoma. Given the limited chemotherapeutic options available for hepatocellular carcinoma, the patient received one cycle of hepatic arterial infusion chemotherapy followed by targeted immunotherapy. CIK cell therapy was administered as an immunomodulatory intervention to enhance the immune function. Following infection control during the initial diagnostic phase, the patient demonstrated improved hematological parameters, including decreased neutrophil counts and recovered lymphocyte levels (**Figure 7A**). Clinically, the patient reported significant symptomatic improvement, including markedly increased appetite and enhanced physical activity. Imaging revealed decreased density of primary lesions with no progression of ascites, although new hepatic lesions were detected (**Figures 6A, B**). Given the patient’s advanced hepatocellular carcinoma diagnosis with severe cachexia at presentation, cancer-related wasting syndrome (cachexia) emerged by week 4 of CIK therapy (week 8 post-diagnosis), potentially compromising hematopoietic function as evidenced by progressive declines in leukocyte, neutrophil, and lymphocyte counts. Concurrently, total protein levels decreased below the lower normal limit (**Figure 8A**). While sustained reduction and stabilization of liver function parameters and tumor markers (**Figures 8A-C**, **9A-C**) indicated controlled tumor progression, these findings did not reflect intact immune competence. The observed leukopenia (reaching nadir values in **Figure 7A**) rather suggested potential immune exhaustion during this clinical phase. During later phases of cellular therapy, a rapid rebound of bilirubin levels was observed, potentially attributable to bile duct compression and edema, which was ultimately discontinued owing to disease progression at the primary site, subsequent worsening of secondary infections, and consequent intolerance to further cell infusions (24). Given the generally compromised physical condition of cancer patients and the risk of disease dissemination, we recommend that allogeneic CIK cell donors be selected from adult first-degree relatives (25). Donor characteristics, including age, sex, and immune status, demonstrated natural variations, resulting in slight differences in CIK cell surface markers (**Supplementary Figure 2**) and required culture duration. Immunophenotyping analysis revealed that CIK cells primarily constituted a heterogeneous population dominated by CD3+ T cells, although some donor-derived cultures showed predominant NKT cell populations (CD3+CD16+CD56+) populations. Current clinical evidence has not identified significant differences in therapeutic efficacy between these cellular composition variants (4).

Many patients require adjuvant chemotherapy postoperatively; however, a subset of them fails to derive long-term benefits due to chemotherapy intolerance, adverse effects, or drug resistance, even after multiple lines of chemotherapy regimens. Although targeted therapies and immune checkpoint inhibitors have addressed the unmet needs in post-chemotherapy treatment, they do not inherently enhance patients’ immune competence. Notably, the efficacy of PD-1/PD-L1 inhibitors depends on the presence of sufficient immune cells (26). Adoptive immune cell therapy offers a novel therapeutic approach by directly augmenting immunity, particularly by enhancing the quantity and quality of T lymphocytes, thereby providing a tangible and effective strategy.

## Limitation and outlook

These three cases represent individual clinical experiences without generalizability. In Case 1, the concurrent use of multiple therapeutic modalities beyond cellular therapy (including targeted and immunotherapies) may have confounded the assessment of CIK treatment efficacy. Cases 2 and 3 reflect more typical clinical scenarios where disease progression could not be fully controlled, ultimately leading to patient demise from various complications such as refractory infections. Notably, all three patients demonstrated consistent symptomatic improvements following CIK cell infusion, including enhanced sleep quality, improved physical activity tolerance, and increased appetite. Importantly, these benefits were achieved without the severe adverse effects typically associated with chemotherapy, resulting in significant quality of life improvements and substantial psychological benefit for the patients.

The final CIK cell products demonstrate considerable variability, potentially attributable to multiple factors including donor health status, laboratory protocols, and even consumable/reagent differences, making standardized quality assessment particularly challenging. These production inconsistencies, coupled with high manufacturing costs and difficulties in product pricing, pose significant obstacles to quality regulation. Regarding cell sources, three options currently exist: autologous, allogeneic, and haploidentical. Allogeneic sources carry potential graft-versus-host disease (GVHD) risks and lack familial connection, while autologous derivation from cancer patients presents concerns about possible tumor cell contamination and frequently yields functionally impaired lymphocytes with poor expansion capacity. The haploidentical approach, utilizing 50mL blood donations from first-degree relatives (≤65 years without blood-borne diseases) offers dual advantages: minimal donor burden and inherent emotional connection. Our observations reveal significant inter-donor variability in cellular composition of final CIK products. Implementing more systematic donor PBMC characterization - particularly through advanced techniques like mass cytometry-could enable deeper correlation analyses between product characteristics and recipient outcomes.

The development of CIK cells derived from induced pluripotent stem cells (iPSCs) and mesenchymal stem cells (MSCs) represents a promising avenue to overcome current limitations, potentially yielding products with minimized GVHD risk and enhanced manufacturing consistency. Establishing hospital-based GMP-compliant laboratories would facilitate immediate clinical application of manufactured CIK products, significantly improving therapeutic accessibility. To fully elucidate the clinical potential of CIK therapy, well-designed prospective studies evaluating combination regimens with chemotherapy, targeted agents, and immunotherapies are essential-requiring (1) rigorous protocol optimization, (2) seamless interdisciplinary collaboration between laboratory and clinical teams, and (3) robust institutional and regulatory support to ensure study validity and patient safety. These coordinated advancements in cell sourcing, production infrastructure, and clinical research methodology will be critical for realizing the full therapeutic potential of CIK cell immunotherapy.

## Conclusion

We recommend the early postoperative application of CIK cell therapy for solid tumor patients, particularly in cases involving: (1) cancer types with limited chemotherapeutic options, (2) chemotherapy-intolerant patients, (3) cases of chemotherapy failure, and (4) patients who have completed standard chemoradiotherapy regimens. The active consideration of CIK therapy with personalized treatment protocols may yield clinically significant outcomes. Regarding the inherent variability of CIK cell products, ongoing advancements in cell culture technologies are progressively addressing these individual differences. Emerging techniques are expected to achieve greater consistency in cellular subtype proportions and absolute cell numbers across different preparations.

## Data Availability

The original contributions presented in the study are included in the article/**Supplementary Material**. Further inquiries can be directed to the corresponding authors.
