# Supplementary material for: Case Report: Three cases with allogeneic CIK therapy against solid tumors
Source: Front Immunol. 2025 Sep 4;16:1658864. doi: 10.3389/fimmu.2025.1658864 (PMC12443832; doi:10.3389/fimmu.2025.1658864)
Supplement: Supplementary file 1 [file DataSheet1.docx]

**Abbreviation**

| AFP | alpha fetoprotein |
| --- | --- |
| ALP | alkaline phosphatase |
| ALT | alanine aminotransferase |
| AST | aspartate aminotransferase |
| CA- | carbohydrate antigen- |
| CEA | carcinoembryonic antigen |
| CIK | cytokine-induced killer |
| CTL | cytotoxic T lymphocyte |
| CYFRA211 | cytokeratin 19 fragment |
| **DPBS** | Dulbecco’ s Phosphate-Buffered Saline |
| **ECG** | **Electrocardiography** |
| GGT | gamma-glutamyl transpeptidase |
| **GMP** | Good Manufacturing Practice |
| **GVHS** | graft-versus-host disease |
| HSA | human serum albumin |
| IFN | interferon |
| IL- | interleukin- |
| iPSC | induced pluripotent stem cell |
| MSC | mesenchymal stem cells |
| NLR | neutrophil-to-lymphocyte ratio |
| NK | natural killer |
| NKT | natural killer T cell |
| NRS | Numerical Rating Scale |
| NSE | neuron specific enolase |
| PBMC | peripheral blood mononuclear cells |
| PET-CT | Positron Emission Tomography-Computed Tomography |
| PFS | progression free survival |
| PD-1/PD-L1 | programmed death-1/programmed death ligand 1 |
| SCC | squamous cell carcinoma antigen |
| SD | stable disease |
| Th | T helper |
| TNF | tumor necrosis factor |

**Laboratory Data**

| **Variable** | **Reference range，adult** |
| --- | --- |
| Leukocytes (per L) | 3.50-9.50 |
| neutrophils (per L) | 1.80-6.30 |
| lymphocytes (per L) | 1.10-3.20 |
| Neu/lym | 2-3.5 |
| T cell (CD3+ %) | 56.00-86.00 |
| Th (CD3+CD4+ %) | 33.00-58.00 |
| CTL (CD3+CD8+ %) | 13.00-39.00 |
| CD4+/CD8+ | 0.71-2.78 |
| NK CD3-CD (16+56) + % | 5.00-26.00 |
| Total proteins (g/L) | 65-85 |
| albumin (g/L) | 35-52 |
| globulin (g/L) | 20-40 |
| ALT (U/L) | Male 9-50/Female 7-40 |
| AST (U/L) | Male 15.0-45.0/Female 13.0-40.0 |
| GGT (U/L) | Male 10.0-60.0/Female 7.0-45.0 |
| ALP (U/L) | 30-120 |
| Total bilirubin (umol/L) | Male 0-26.0/Female 0-21.0 |
| Direct bilirubin (umol/L) | Male 0-3.4/ Female 0-4.00 |
| CA72-4 (U/ml) | ＜6.90 |
| CA125 (U/ml) | ＜35 |
| CA199 (U/ml) | ＜23 |
| Ferritin (ng/ml) | 30.1-400.2 |
| CEA (ng/ml) | ＜5 |
| CYFRA211 (ng/ml) | 0-3.30 |
| NSE (pg/ml) | ＜14.38 |
| SCC (ng/ml) | ＜1.65 |
| AFP (ng/ml) | 0-7 |
| IL-1β(pg/ml) | 0-12.1 |
| IL-2 (pg/ml) | 0-11.4 |
| IL-4 (pg/ml) | 0-12.9 |
| IL-5 (pg/ml) | 0-3.4 |
| IL-6 (pg/ml) | 0-20 |
| IL-8 (pg/ml) | 0-21.4 |
| IL-10 (pg/ml) | 0-5.9 |
| IL-12p70 (pg/ml) | 0-3.2 |
| IL-17 (pg/ml) | 0-20.6 |
| IFN-α (pg/ml) | 0-7.9 |
| IFN-γ (pg/ml) | 0-17.3 |
| TNF-α (pg/ml) | 0-5.5 |

**CIK Culture**

**The cell culture process was conducted in a GMP-compliant laboratory. On Day 1, primary cell isolation was initiated by collecting at least 50 ml of peripheral venous blood from first-degree relatives, followed by centrifugation at 3,000 rpm for 8 min. The supernatant plasma was transferred to a new 50 mL tube, labeled as "Plasma," and heat-inactivated at 56°C for 30 min. The remaining blood cells were carefully layered over Ficoll-Paque™ (GE #17-1440-03, clinical grade) in a 15 ml tube and centrifuged at 2,000 rpm (slow acceleration/deceleration) for 20 min. The buffy coat was aspirated into a new 50 ml tube, resuspended in complete medium (TAKARA, #GT-T551H3), and centrifuged at 2,000 rpm for 5 min marked as “Cells”. The "Plasma" tube supernatant was retained (4°C storage), while the "Cells" tube pellet was preserved. cells were seeded into a T75 flask (Corning, #430641) containing culture medium supplemented with recombinant human IL-2 (1 million IU, Jiangsu Jinsili) and CIK expansion factors (MDSS^®^, #MDL1501), which the main ingredients are IFN-γ and anti-CD3, followed by incubation at 37°C with 5% CO₂. Medium replenishment, expansion, and inactivated-plasma supplementation were performed on days 4, 6, and 8, with cells transferred to culture bags (TAKARA, #GT-T610) as needed. On Day 11, a 5ml aliquot was sent for sterility testing (bacteria, fungi, mycoplasma, endotoxins, and pyrogens). On Day 12, cells were harvested by centrifugation (2,000 rpm, 5 min), washed twice with saline, and resuspended in 250 ml of 1% HSA-supplemented saline for transfusion bag (Suzhou LAISHI, 250ml) transfer. A 2 ml sample was retained for viability, count, and surface marker analysis, ensuring compliance with GMP standards. Representative microscopic images of CIK (Supp. Figure 1).**


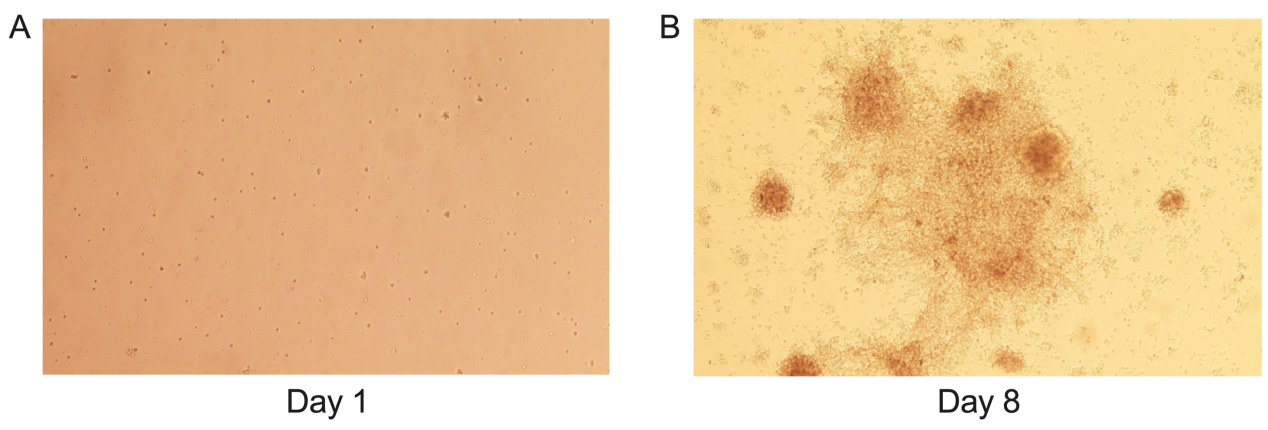
**Supp. Figure 1** Representative microscopic CIK (*×100*) in Day1 and Day 8.

**Flow Cytometry Analysis of Cell Surface Markers**

**The cells were washed once with DPBS (TAKARA, #FU0021), centrifuged at 2,000 rpm for 5 min, and the supernatant was discarded. Approximately 1×10⁶ cells per sample were stained with a fluorescent antibody (Beckman, CD45-KRO, #B36294; CD3-FITC, #IM1281U; CD4-APC #IM2468; and CD8-PC5.5 #B21205, CD3-FITC/CD (16+15)-PE, #A07735) according to the manufacturer’s recommendations, with an unstained control prepared for background correction. After adding an appropriate volume of antibody dilution buffer, the samples were vortexed thoroughly and incubated for 20 min at room temperature in the dark. Following incubation, the cells were washed again with DPBS, centrifuged at 2,000 rpm for 5 min, and the supernatant was removed. Finally, the cells were resuspended in 200 μL of DPBS, filtered through a cell strainer (Falcon, #352360), and immediately analyzed by flow cytometry. Representative surface marker of CIK transfusion products with analyzed by flow cytometry (Supp. Figure 2).**

**
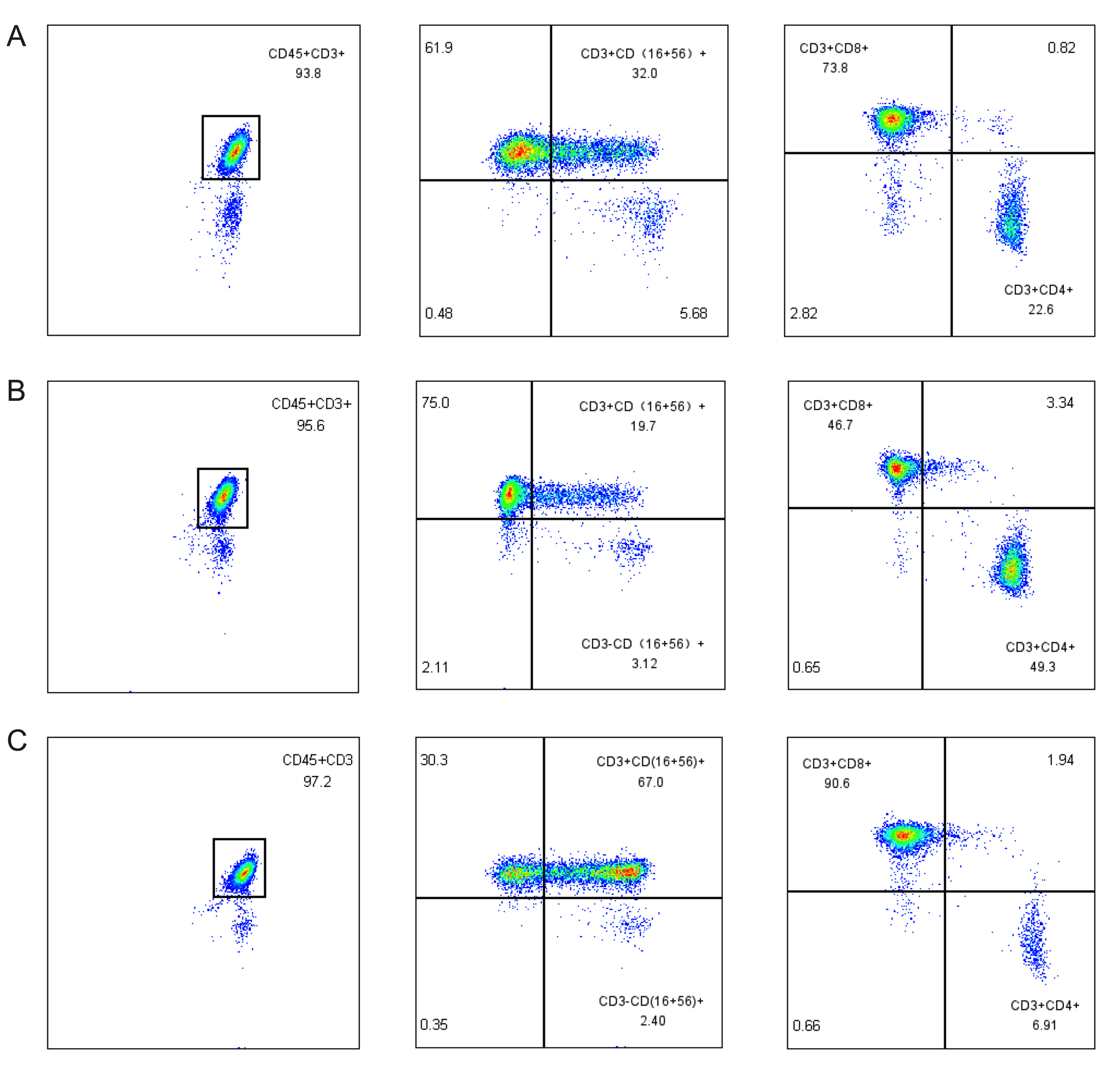
**

**Supp. Figure 2** Representative surface marker of CIK transfusion product from Case 1-3

**CIK Administration and Management for Possible Side Effects**

**The CIK product is a 250 ml/bag mixed solution of normal saline and cells with cell viability ≥90% and a cell concentration of 10-30×10⁶ cells/ml (2.5-7.5×10⁹ cells/bag). Each treatment cycles consisted of two bags administered over two consecutive days (one bag per day) and repeated every 2-4 weeks. For infusion, a blood transfusion set should be used to prevent clogging, with 50 ml normal saline pre-flush followed by a 250 ml cell product initially infused at a slow drip rate for the first 10 minutes for patient monitoring, and then increased to a faster administration within 60-90 min, followed by a 50 ml normal saline post-flush. Continuous electrocardiography (ECG) monitoring was performed throughout the procedure. The cell product can be stored at 4-8°C for up to 12 h but must be equilibrated to room temperature prior to infusion.**

**Common adverse reactions associated with CIK cell infusion include fever, chills, somnolence, fatigue, malaise, and dizziness, most of which are transient and do not require specific treatment. Fever typically occurs 4-8 h post-infusion and usually resolves spontaneously within 2-4 h; antipyretics (e.g., acetaminophen 500 mg q12h p. o.) may be administered if body temperature exceeds 39°C. For rare mild anaphylaxis, promethazine (25 mg q8h p. o.) is recommended, whereas severe cases may require hydrocortisone (100 mg once ivgtt).**
